# Metaphor Comprehension in Low and High Creative Individuals

**DOI:** 10.3389/fpsyg.2018.00482

**Published:** 2018-04-09

**Authors:** Yoed N. Kenett, Rinat Gold, Miriam Faust

**Affiliations:** ^1^Department of Psychology, University of Pennsylvania, Philadelphia, PA, United States; ^2^The Leslie and Susan Gonda (Goldschmied) Multidisciplinary Brain Research Center, Bar-Ilan University, Ramat Gan, Israel; ^3^Department of Communication Disorders, Sackler Faculty of Medicine, Tel Aviv University, Tel Aviv, Israel; ^4^Department of Psychology, Bar-Ilan University, Ramat Gan, Israel

**Keywords:** creativity, metaphor comprehension, novel metaphors, conventional metaphors, semantic memory

## Abstract

The comprehension of metaphors involves the ability to activate a broader, more flexible set of semantic associations in order to integrate the meanings of the weakly related parts of the metaphor into a meaningful linguistic expression. Previous findings point to a relation between levels of creativity and efficiency in processing metaphoric expressions, as measured by reaction times (RTs) and error rates. Furthermore, recent studies have found that more creative individuals exhibit a relatively more flexible semantic memory structure compared to less creative individuals, which may facilitate their comprehension of novel metaphors. In the present study, lower and higher creative individuals performed a semantic relatedness judgment task on word pairs. These word pairs comprised four types of semantic relations: novel metaphors, conventional metaphors, literal word pairs, and meaningless word pairs. We hypothesized that the two groups will perform similarly in comprehending the literal, unrelated, and the conventional metaphoric word pairs. However, with respect to novel metaphors, we predicted that higher creative individuals will demonstrate better performance compared to lower creative individuals, as indicated by smaller RTs and more accurate responses. Our main finding shows that higher creative individuals were faster in comprehending both types of metaphors, conventional and novel, compared to lower creative individuals. Furthermore, higher creative individuals were significantly more accurate than lower creative individual only in comprehending novel metaphors. The findings are discussed in light of previous findings regarding the relation between metaphor comprehension, semantic memory, and creativity.

## Introduction

The creative processes involved in producing higher order linguistic outputs such as irony, humor, and metaphors include linguistic flexibility, fluency, and originality (Faust, 2012; Mirous and Beeman, 2012). These higher order linguistic outputs all share the need to process, activate, and maintain multiple meanings of a concept, also including uncommon and weakly related meanings (Cushen and Wiley, 2011). As such, semantic creativity is achieved by combining seemingly unrelated or distantly related concepts to create a meaningful linguistic expression (Mednick, 1962; Kenett, in press). Surprisingly, only a scarce amount of studies examined how individual differences in creative ability relate to processing high-order language products, like metaphors (e.g., Gold et al., 2011). This is the aim of the current study.

Metaphors are extremely common in language (Lakoff and Johnson, 1980). This is due to their ability to efficiently express ideas that would be awkward to describe literally (Glucksberg, 2001). Metaphors are composed from a topic, the subject to which attributes are ascribed, and the vehicle, the object whose attributes are borrowed. The comprehension of metaphors (CoM) requires activating a broader, more diffuse range of semantic associations to enable the combination of weakly related concepts to a novel and appropriate metaphoric expressions (Bowdle and Gentner, 2005). However, with its continued usage, novel metaphors become conventionalized, as their metaphorical meaning becomes integrated within semantic memory structure (Glucksberg, 2003; Bowdle and Gentner, 2005; Mashal and Faust, 2009; Faust, 2012; Goldstein et al., 2012). Thus, the comprehension of conventional metaphors is based on the retrieval of the meaning of the metaphor, whereas the comprehension of novel metaphors is based on the creation of new meaning. This difference between the processes involved in the comprehension of conventional and novel metaphors is described in the career of metaphor hypothesis (Bowdle and Gentner, 2005). According to this hypothesis, different cognitive processes underlie the comprehension of conventional and novel metaphors. Similarly, Gentner’s structural mapping theory (1983) describes the different cognitive processes that underlie the comprehension of the two metaphor types. According to the structural mapping theory, novel metaphors are comprehended by establishing a correspondence between the concepts of the topic and vehicle (Gentner, 1983). As a metaphor is frequently used and becomes conventionalized, its processing becomes based on a categorization process. Such a categorization process is more rapid and less demanding than the comparison process, but requires an existing metaphoric category to allow it. Thus, according to the career of metaphor model, as a novel metaphor becomes more conventionalized, its comprehension process shifts from a comparison process to a categorization process (Gentner and Bowdle, 2001).

The findings of several studies to date suggest that although both conventional and novel metaphors involve some kind of semantic violation, they may be regarded as distinct linguistic expressions which involve different semantic processing mechanisms (Giora, 1997; Bowdle and Gentner, 2005; Faust, 2012; Mirous and Beeman, 2012). Such a distinction has also been shown at the neurocognitive level, both in typical (Arzouan et al., 2007b; Lai et al., 2009; Goldstein et al., 2012; Lai and Curran, 2013; Mashal et al., 2015) and atypical (Gold and Faust, 2010, 2012; Zeev-Wolf et al., 2014, 2015) populations. Under this framework, conventional metaphors are comprehended based on the pre-established, salient semantic relations in memory between the individual words constituting the conventional metaphor (Bowdle and Gentner, 2005). Novel metaphors, however, require establishing new connections between concepts in memory, a process also related to creative thinking (Mednick, 1962). Thus, semantic memory structure plays a critical role in CoM, and its investigation can shed further light on the cognitive processes involved in metaphor comprehension. One approach to studying semantic memory structure is via computational modeling (McRae and Jones, 2013; Jones et al., 2015).

A comprehensive computational model that can characterize the semantic processing between the topic and the vehicle of a metaphor is the predication model (Kintsch, 2000, 2001, 2008; Kintsch and Bowles, 2002; Al-Azary and Buchanan, 2017). This model is composed of two components: a computational representation of the meanings of words and the application of these representations in computing a contextually appropriate interpretation of statements (Kintsch, 2000). To model the meaning of a metaphor, a spreading activation-based semantic network is constructed. In this semantic network, the semantic neighborhood of the vehicle is examined for words that also happen to be related to the topic, while inhibiting those that are unrelated. According to this process, semantic neighbors that are related to both the topic and the vehicle are strengthened whereas other neighbors of the individual words are inhibited. The semantic neighbors that are activated (i.e., the semantic neighbors of the vehicle which are also related to the topic) along with the topic and vehicle are then used to compute a vector which is taken to represent the metaphor’s meaning (Al-Azary and Buchanan, 2017). Thus, the model can predict performance in comprehension of different metaphor types, based on their novelty (Kintsch, 2008).

Based on this predication model, Chiappe and Chiappe (2007) examined the role of working memory and specifically inhibitory control in processing metaphors. The authors show how individual differences in inhibitory control and vocabulary knowledge were related to metaphor comprehension. Thus, the authors concluded that metaphor processing depends on fluid and crystallized cognitive abilities (Chiappe and Chiappe, 2007). These findings can be related to the predication model of metaphor comprehension in two ways. First, individuals with low inhibitory control processes may not have the resources required to inhibit irrelevant, salient, semantic properties. Second, such individuals may have a semantic network structure that is not rich enough to create semantic neighborhoods that are large enough to include properties pertinent to the interpretation of a metaphor (Chiappe and Chiappe, 2007). Thus, individual differences in semantic memory structure are related to proficiency in CoM in general and specifically to differences in the processing of novel versus conventional metaphors.

In the past few years, several studies have applied network science methodologies to examine the possibility that higher creative people have a more flexible semantic memory structure than lower creative people (see Kenett, in press for a review). Network science is based on mathematical graph theory, providing quantitative methods to investigate complex systems as networks (Borge-Holthoefer and Arenas, 2010; Baronchelli et al., 2013; De Deyne et al., 2016; Karuza et al., 2016). Kenett et al. (2014) applied network science methods to compare the structure of semantic memory in low and high creative individuals. In accordance with the associative theory of creativity (Mednick, 1962), the authors found that the semantic network of higher creative participants was more flexible than that of lower creative participants, demonstrating higher connectivity and lower global distances between concepts in their semantic network (Kenett et al., 2014; see also Kenett et al., 2018). These findings were then replicated in an independent between-subject study (Kenett et al., 2016) and partially replicated in a within-subject design (Benedek et al., 2017).

Since the comprehension of novel metaphors requires the recognition or construction of non-salient connections between words in order to integrate their meanings and create plausible expressions, novel metaphor processing may be related to individual differences in creative ability. The associative theory of creativity posits that individual differences in creativity relate to differences in semantic memory structure, which facilitates novel combinations between distant or weakly connected concepts (Mednick, 1962; Kenett, in press). Taken together with the predication model of metaphor comprehension (Kintsch, 2000), semantic memory structure may link individual differences in creativity with metaphor comprehension. However, the link between metaphor comprehension and creative ability via semantic memory structure has not been directly examined.

To date, the main empirical study that examined this relation was conducted by Gold et al. (2011), who examined the relation of performance on an offline measure of creative thinking – the remote association test (RAT; Mednick, 1962) – and comprehension of different types of word pairs, processed by the two cerebral hemispheres (Gold et al., 2011). The authors show that the performance on the remote association task was significantly negatively correlated with reaction times (RTs) when attempting to comprehend conventional and novel metaphors. However, Gold et al.’s (2011) study examined the relation between performance on a metaphor comprehension task and general performance on a creative task. In the current study, we examine how low and high creative individuals perform in a CoM task (Faust, 2012). Thus, this study aims to replicate and extend the findings of Gold et al. (2011), by examining performance on the CoM task in predefined groups of low and high creative individuals. We predicted that high creative individuals will perform better (lower RT and higher accuracy) in processing novel metaphors, compared to low creative individuals. In addition, we do not expect to find any significant differences between the two groups in processing conventional metaphors, literal or unrelated word pairs.

## Materials and Methods

### Participants

Participants were recruited from a larger sample that was part of a study on individual differences in creative ability (Kenett et al., 2014). These individuals performed a battery of creativity measures, which included the Hebrew version of the RAT (Mednick, 1962; Nevo and Levin, 1978), and the Hebrew version of the Wallach and Kogan battery of creativity tests (Wallach and Kogan, 1965; Milgram and Milgram, 1976). These creativity measures were used, based on a decision tree approach (Kopiez et al., 2006), to classify participants into low semantic creative (LSC) and high semantic creative (HSC) groups, consisting of 33 participants each (see below). One participant was removed from each group due to low accuracy rate (<0.5) on the literal meaning condition (see below). The remaining 32 participants in the two groups were matched for age, years of education, intelligence (measured with the Raven Standard Progressive Matrices Short Version; Van der Elst et al., 2013) and handedness (measured with the Edinburgh Handedness Inventory; Oldfield, 1971) scores (**Table 1**). All participants were Hebrew native speakers with normal or corrected to normal vision. Participants received 80 ILS for participation in this study. This study was approved by the Bar-Ilan university internal review board.

**Table 1:** Low semantic creative (LSC) and high semantic creative (HSC) group details (*SD* in parentheses).

Parameter	LSC	HSC
*N*	32 (14/18)	32 (6/26)
Age	23.4 (2.4)	22.7 (2.2)
Education	14.1 (1.6)	13.8 (1.5)
EHI	92.5 (9.2)	90.6 (9.6)
RSPM-SV	111.5 (8.5)	114.3 (9.0)
TACT_F	65.9 (15.7)	88 (24.0)
TACT_Q	34.0 (12.5)	50.4 (21.1)
RAT	7.1 (2.7)	13.2 (3.0)

*N, the number of participants in each group (male/female), age, mean group age in years, education, mean education years; EHI, mean Edinburgh Handedness Inventory score; RSPM-SV, mean Raven Standard Progressive Matrices Short Version score; TACT_F, mean TACT fluency score; TACT_Q, mean TACT quality score; RAT, mean RAT score*.

### Materials

#### Assessing Creative Ability

##### Remote association test

The RAT (Mednick, 1962) examines individual differences in creative ability. In this test, participants are presented with a triplet of seemingly unrelated words (e.g., *Cottage*, *Swiss*, and *Cake*) and are required to find a single fourth word that is related to each of these words (e.g., Cheese; Bowden and Jung-Beeman, 2003). This task is accepted as examining semantic creativity and has been empirically widely used for its investigation (Gold et al., 2011; Storm et al., 2011; Mirous and Beeman, 2012). In our research, we used the Hebrew version of the RAT (Nevo and Levin, 1978), which contains 25 triplets with varying degree of difficulty and lasts 15 min. The RAT score is the sum of correct answers given by the participant.

##### Tel Aviv University creativity test (Milgram and Milgram, 1976)

This test is a modified Hebrew version of the Wallach and Kogan (1965) battery of divergent thinking tests (Runco and Acar, 2012), frequently used in creativity research (Baird et al., 2012). The Tel Aviv University creativity test (TACT) measures verbal and visual creativity by producing two scores – fluency (number of responses provided) and quality (originality and applicability of response). The test is composed of four sub-tests – two verbal (alternative uses and pattern matching) and two visual (similarities and line meanings). Each sub-test lasts 6 min and includes four open questions. The fluency score was calculated by counting the number of different answers. The quality score was determined by three independent judges (inter-rater agreement > 0.8) who evaluated the originality, on a Likert scale ranging from 1 (low originality) to 3 (high originality) and applicability, on a Likert scale ranging from 1 (low applicability) to 4 (high applicability) of unique responses only – answers that appeared in only 5% or less of the sample. The originality and applicability ratings were then transformed into a quality scale ranging from 0 (no creative quality) to 10 (extreme creative quality). Finally, a participant’s quality score was computed by counting their responses that scored 3 or above on the quality scale (for more details, see Milgram and Milgram, 1976). Thus, for each participant, fluency and quality scores were computed for each sub-test, as well as an averaged general fluency and quality scores.

##### Classifying participants into creativity groups

Participant’s performance on the RAT and TACT was used to classify the participants into LSC and HSC groups. Some studies have argued that the RAT and divergent thinking tasks such as the TACT measure different creative abilities (Lee and Therriault, 2013; Lee et al., 2014). However, other studies have shown that the RAT involves an initial divergent thinking stage (Taft and Rossiter, 1966; Smith et al., 2013; Smith and Vul, 2015). For example, Smith et al. (2013) analyzed the guesses participants generated while solving the RAT. The authors show how performance in the RAT involves two stages – an initial divergent thinking stage of generating guesses and an evaluation stage of these guesses. Thus, we classify participants into LSC and HSC based on how their TACT scores predict their performance on the RAT.

This was achieved via a decision tree approach, which is a statistical method for analyzing multivariate data (Lafond et al., 2009; Galimberti and Soffritti, 2011; Brandmaier et al., 2013). A decision tree attempts to predict based on independent variables (different measures of the TACT) specific classes of a dependent variable (all participants who received a certain score on the RAT). The dependent variable can be split into smaller and smaller classes (branches), until specific stopping rules are achieved (Galimberti and Soffritti, 2011; Brandmaier et al., 2013). Thus, this method strives to find clusters that represent a sufficient range of the dependent variable and are separable with an accepted error (Kopiez et al., 2006). This method derives decisions, or classification rules, which form the different branches of the tree. To classify the participants to the LSC and HSC groups, we applied the JMP software^1^ classification and regression tree approach (Galimberti and Soffritti, 2011), to predict performance in the RAT based on the different TACT fluency and quality scores. In this sense, this approach computes classification rules on how different ranges and combinations of the different TACT scores predict participants’ performance on the RAT. We sorted these classification rules in an ascending fashion, from predicting the lowest to the highest RAT scores. Participants positioned in the lower tertile of these classification rules were considered as LSC and participants positioned in the higher tertile as HSC, leading to groups of 33 participants in each group (Kenett et al., 2014). Thus, participants were independently assigned into the two groups, based on their performance in creativity tasks and then their performance in the CoM task was examined.

#### Comprehension of Metaphors

In this task (Faust, 2012), participants are presented with 240 two-word noun–noun or adjective–noun combinations in Hebrew, which can either have a literal [literal word pairs (LPs); *burning fire*, *problem resolution*], conventional metaphoric [conventional word pairs (CPs); *lucid mind*, *transparent intention*], and novel metaphoric [novel word pairs (NPs); *ripe dream*, *conscience storm*] meaning or are unrelated (UPs: *indirect blanket*, *wisdom wash*). The stimuli were identical to those used in previous studies (Gold and Faust, 2010; Gold et al., 2010), and participants are asked to decide whether the two words are related to each other or not (Faust, 2012). Two-word metaphors were used, in order to avoid the confounding effects of sentence level processes or larger context (Faust et al., 2003, 2006). Furthermore, the novel metaphorical expressions were taken from original Hebrew poetry and thus had high ecological validity and were, at least potentially, meaningful.

The stimuli were similar to those used in previous experiments (Arzouan et al., 2007a,b; Faust and Mashal, 2007; Gold et al., 2010). All primes were nouns and both prime and target words consisted of two to six letters. Word length was counterbalanced across the four types of word pairs. Thus, each condition contained equal numbers of two, three, four, five, and six letter primes and targets. Stimuli were also balanced between conditions according to word frequency, concreteness, grammatical category, and syntactic structure. Several pretests were performed to determine the type of semantic relationship between the two words in each pair, concreteness and word frequency. The aim of the first pretest was to determine the type of each two-word expression (metaphoric, literal, or unrelated). In order to do so, 40 judges, who did not participate in this study, were presented with a list of two-word expressions and asked to decide whether each expression is literally plausible, metaphorically plausible, or not plausible. Expressions that were rated by at least 80% of the judges as either metaphorically/literally plausible or not plausible were selected as expressions with either a metaphoric or a literal meaning or as unrelated word pairs, respectively.

In order to distinguish between unfamiliar novel metaphors and conventional metaphors, another group of 35 judges, who did not also participate in this study, were presented with a list of only the plausible metaphoric expressions from the first pretest. Participants were asked to rate the degree of familiarity of each metaphoric expression on a five-point familiarity scale ranging from 1 (highly non-familiar) to 5 (highly familiar). Metaphoric expressions scoring less than 2.4 on the familiarity scale were selected for the study as novel metaphors (rating average = 1.53, *SD* = 0.23), whereas those scoring above 3.6 on this scale were selected as conventional metaphors (rating average = 4.45, *SD* = 0.44). The degree of familiarity of these two types of metaphors was significantly different, *t*(118) = 45.72, *p* < 0.001.

In another pretest, 23 additional judges were presented with the list of all primes and targets and were asked to rate the level of concreteness on a scale ranging from 1 (highly abstract) to 5 (highly concrete). Words with an average of less than 3 (on the 1–5 scale) were considered as abstract words whereas words with an average of more than 3 were considered concrete words. For the prime words, 70%, 75%, 71.66%, and 76.66% of the words were judged as concrete for the novel metaphors (*M* = 3.9, *SD* = 0.57), conventional metaphors (*M* = 4.2, *SD* = 0.52), literal (*M* = 4.1, *SD* = 0.56), and unrelated (*M* = 4.2, *SD* = 0.54) conditions, respectively. For the target words 65%, 68.33%, 63.33%, and 65% of the words were judged as abstract for the novel metaphors (*M* = 2.1, *SD* = 0.42), conventional metaphors (*M* = 2.3, *SD* = 0.39), literal (*M* = 2.2, *SD* = 0.37), and unrelated (*M* = 2.1, *SD* = 0.44) conditions, respectively.

Since in Hebrew there is no extensive database for word frequency, the fourth pretest tested the word frequency. Forty-five additional judges, who did not participate in the former pretests and not in the experiment, were presented with the list of all the words and asked to rate their degree of frequency on a five-point frequency scale ranging from 1 (highly non-frequent) to 5 (highly frequent). The average rates on the frequency scale for the target words were 3.57, 3.59, 3.65, and 3.62, for the novel metaphors, conventional metaphors, literal, and unrelated, respectively. The average rates on the frequency scale for the priming words were 3.74, 3.60, 3.68, and 3.72 for the novel metaphors, conventional metaphors, literal, and unrelated, respectively. No significant difference was found for the target and the priming words between the four conditions (*F* < 1).

### Procedure

Participants signed a consent form, and then they were instructed on the task and given examples of all four types of word-pair relations. Participants sat 50 cm from a CRT screen. The task was conducted using the E-prime software (Schneider et al., 2002). The stimuli were presented using white letters against a black screen in the following time sequence: fixation cross (200 ms), first word (200 ms), blank screen (400 ms), and second word (200 ms). Participants were instructed to judge whether the presented two words were related to each other or not. Participants were instructed to use their right hand to make their decision, using the index and middle fingers to indicate related and unrelated decisions. Once the participant pressed the button, the next trial was immediately initiated. Stimuli presentation was randomized and the relation between keyboard keys and decision (related or unrelated) was counter balanced across participants. The task included a short practice composed of four examples of each word-pair relation that was not used in the task itself. Participants were instructed to respond as quickly and as accurately as they could.

## Results

**FIGURE 1:**
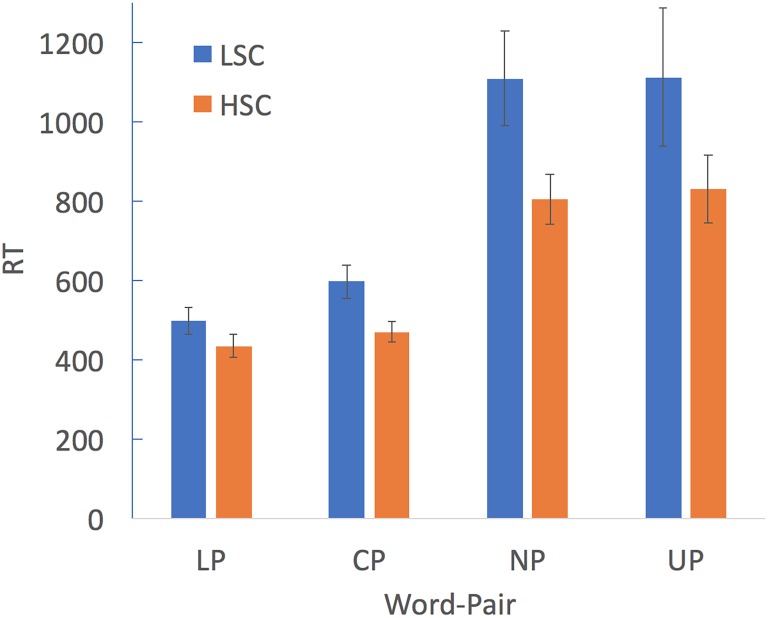
RT of performance on the comprehension of metaphors task for the LSC and HSC groups. Error bars denote SE.

Trials in which RT was lower than 150 ms were removed. In addition, for each participant, trials which were above or below 2.5 *SD* for each condition were also deleted from the final data analysis. RT analysis was conducted only for correct responses. A group (LSC, HSC) X word-pair type (LP, CP, NP, and UP) mixed-design ANOVA was conducted in order to examine the effect of word-type on participants’ CoM RT (**Table 2** and **Figure 1**). This analysis revealed a significant main effect of group, *F*(1,62) = 4.305, *p* = 0.042, *η*^2^ = 0.065. This main effect resulted from the HSC group having shorter RTs (*M* = 635 ms, *SD* = 253 ms) than the LSC group (*M* = 830 ms, *SD* = 466 ms) in the CoM task (*p* < 0.04). This analysis also revealed a significant main effect of word-pair type, *F*(3,186) = 33.864, *p* < 0.001, *η*^2^ = 0.353. *Post hoc t*-test analysis (corrected for multiple comparisons) revealed that this effect is driven by a slowing of average RT as the relation between the word pairs changes from literal to unrelated (LP: *M* = 496 ms, *SD* = 187 ms; CP: *M* = 566 ms, *SD* = 219 ms; NP: *M* = 977 ms, *SD* = 558 ms; and UP: *M* = 981 ms, *SD* = 782 ms) conditions (all *p*’s < 0.001). This analysis did not reveal a significant interaction effect, *F*(3,186) = 1.601, *p* = 0.19, *η*^2^ = 0.025. However, based on our hypotheses and previous studies (Gold and Faust, 2010), we conducted planned contrast *t*-test analysis between the two groups for all word-pair conditions. This analysis revealed differences between both groups in comprehending both conventional (*p* < 0.01) and novel (*p* < 0.02) metaphors (**Table 2**). No significant differences were found for the literal (*p* = 0.12) or the unrelated (*p* = 0.15) conditions.

**Table 2:** RT and accuracy of the different word pairs in the two groups (*SD* in parentheses).

	RT		Accuracy	
	
	LSC	HSC	LSC	HSC
Literal meaning	499 (188)	435 (163)	0.98 (0.03)	0.98 (0.03)
Conventional metaphors	597 (242)	470 (146)	0.93 (0.05)	0.95 (0.04)
Novel metaphors	1110 (678)	803 (360)	0.48 (0.23)	0.62 (0.24)
Unrelated meaning	1113 (989)	831 (489)	0.92 (0.08)	0.90 (0.10)

*LSC, low semantically creative; HSC, high semantically creative*.

**FIGURE 2:**
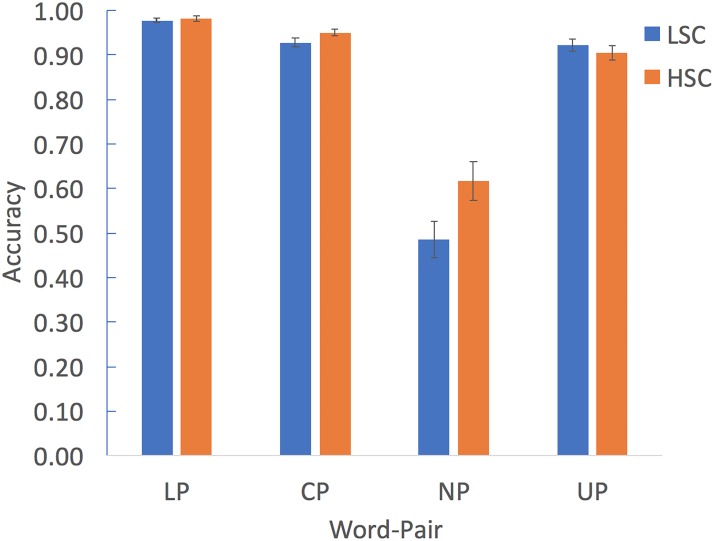
Accuracy of performance on the comprehension of metaphors task for the LSC and HSC groups. Error bars denote SE.

A group (LSC, HSC) X word-pair type (LP, CP, NP, and UP) mixed-design ANOVA was conducted in order to examine the effect of word-type on participants CoM accuracy (**Table 2** and **Figure 2**). This analysis revealed a significant main effect of group, *F*(1,62) = 5.75, *p* < 0.02, *η*^2^ = 0.085. *Post hoc* analysis revealed that overall, the HSC group performed better (*M* = 0.86, *SD* = 0.06) than the LSC group (*M* = 0.83, *SD* = 0.06) on the CoM task (*p* < 0.02). This analysis also revealed a significant main effect of word-type, *F*(3,186) = 141.59, *p* < 0.001, *η*^2^ = 0.695. This effect resulted from a significant lower accuracy for judging NP compared to all other word-pairs (all *p*’s < 0.001 for both groups). Finally, we found a significant interaction between group and word-type on accuracy, *F*(3,186) = 3.99, *p* < 0.007, *η*^2^ = 0.061. This interaction stemmed from a different effect of word-pair type on the accuracy ratings in both groups. *Post hoc t*-test analysis (corrected for multiple comparisons) revealed that the HSC group was significantly more accurate in judging NP (*p* < 0.03) and exhibited a similar trend in judging CP (*p* < 0.07), compared to the LSC group. No significant differences between the two groups were found for the literal (*p* = 0.6) and unrelated (*p* = 0.42) word-pair conditions (**Table 2**).

## Discussion

In the current study, we investigate how lower and higher creative individuals perform on a metaphor comprehension task. Both groups were tested on a CoM task in which they had to decide whether two words were related to each other or not (Faust, 2012). These word pairs either had a literal meaning (e.g., *school-bus*), a conventional metaphor meaning (e.g., *iron-fist*), a novel metaphoric meaning (e.g., *mercy-blanket*), or were unrelated (e.g., *school-sky*). We compared the groups’ RT and accuracy for the different word-type conditions, a common approach in the study of metaphor comprehension (e.g., Hoffman and Kemper, 1987; Kemper, 1989; McElree and Nordlie, 1999; Faust and Mashal, 2007; Faust, 2012).

Our main finding was that higher creative individuals were overall faster in comprehending the different word-types than the lower creative individuals. However, while the higher creative individuals were significantly faster in comprehending conventional and novel metaphor word pairs, they were more accurate only for the novel metaphor condition. These findings replicate a previous study that demonstrated a relation between performance on a creativity task and novel metaphor processing (Gold et al., 2011). The better performance of the higher creative group in processing novel metaphors may be explained in terms of semantic memory structure. It has been suggested that higher creative individuals may be characterized with a higher connected semantic memory structure. Such a structure supports the inhibition of the connections between weak, or remote, concepts (Mednick, 1962; Kenett et al., 2014; Benedek et al., 2017; Hass, 2016, 2017; Kenett, in press). In support of this interpretation, Rossman and Fink (2010) show that high creative individuals judge weakly connected concepts as having stronger relations than low creative individuals.

With regard to conventional metaphors, we hypothesized that no differences between the two groups in RT and accuracy will be found. This hypothesis was only partially supported, by finding that the higher creative group being quicker, but not more successful, in comprehending conventional metaphors than the lower creative group. This may likely be due to the fact that comprehending conventional metaphors is similar to comprehending literal word pairs and relies on different cognitive mechanisms required in comprehending novel metaphoric word pairs (Bowdle and Gentner, 2005; Faust, 2012). Furthermore, previous studies have shown that higher creative individuals are generally quicker in generating responses than lower creative individuals (Mednick et al., 1964; Vartanian et al., 2007, 2009; Benedek and Neubauer, 2013). Such a difference is attributed to the role of internal focused attention, in line with current theories focusing on the role of top-down, executive processes, in creativity (Silvia, 2015; Chrysikou, 2018). However, such an account does not address the differences we found in the successful comprehending novel metaphors.

Another finding of the present study was that in both groups of participants, RTs during the processing of novel metaphors and unrelated word pairs were longer. In other words, as the relation between the word pairs became more distant, comprehending them took longer, evident in higher RTs. Recently, Kenett et al. (2017) examined the relation between increasing semantic distance and relatedness judgments. Using a similar task, the authors show that as semantic distance (computed based on a semantic network) grows, participants’ RT increases and they are less likely to judge word pairs as related. Thus, our findings provide further empirical support linking semantic distance and relatedness judgments. Importantly, our findings and those of Kenett et al. (2017) directly support Clevenger and Edwards (1988), who argued that semantic distance (and thus also semantic similarity) plays an important role in metaphor construction.

According to the career of metaphor theory (Bowdle and Gentner, 2005), novel metaphors require extensive cognitive effort to process, effort that diminishes with increasing conventionalization of the metaphor. Faust (2012) has related this trajectory onto hemispheric differences in language processing, contingent on the semantic relation between word pairs (see also Kenett et al., 2015). According to her view, the left cerebral hemisphere uniquely contributes to the processing of LPs, which involves the retrieval of systematic relations between interconnected concepts in semantic memory. Similar to LPs, conventional metaphoric word pairs are processed based on the retrieval of pre-established, salient semantic relations. Novel metaphors, however, are composed of distant, unusual relations between concepts. The right cerebral hemisphere uniquely contributes to the processing of novel metaphoric word pairs, which involves the creation of new meanings from these unusual relations. This view has been empirically supported by neurocognitive studies both in typical (Lai et al., 2009; Goldstein et al., 2012; Lai and Curran, 2013) and atypical (Gold and Faust, 2010; Gold et al., 2010; Zeev-Wolf et al., 2015) populations. The processing of novel metaphors requires a flexible, higher connected semantic memory structure that facilitates understanding of newly created combinations. Such a flexible semantic memory structure can tolerate semantic violations and cope with multiple, less dominant interpretations. As such, higher successful performance in comprehending novel metaphors should indicate a more flexible semantic memory structure. Thus, the investigation of semantic memory structure via computational models (McRae and Jones, 2013; Jones et al., 2015) has the potential to shed light on metaphor comprehension.

One such computational model that has been proposed in regard of metaphor comprehension is the predication model (Kintsch, 2000, 2001; Chiappe and Chiappe, 2007; Al-Azary and Buchanan, 2017). This computational model assumes that metaphor comprehension is contingent on the activation of semantic neighborhoods in specific context (Kintsch, 2008). Empirically examining the predication model, Chiappe and Chiappe (2007) show how crystallized knowledge and fluid intelligence relate to CoM. While the authors interpret their findings in the context of inhibition of salient properties in a semantic space, their findings can also be interpreted as different semantic memory structures contributing to performance in metaphor comprehension. Furthermore, in the past few years, a growing body of research is applying computational methods to examine how semantic memory structure relates to individual differences in creative ability (Beaty et al., 2014; Kenett et al., 2014, 2016; Hass, 2016, 2017; Benedek et al., 2017; Kenett, in press). Specifically, Kenett et al. (2014) show how the semantic memory structure of higher creative individuals is more flexible, compared to lower creative individuals (see also Kenett et al., 2018). These findings were replicated by an independent group-based study (Kenett et al., 2016) and partially replicated by an individual-based study (Benedek et al., 2017). Importantly, the same participants that took part in the study of Kenett et al. (2014) and exhibited a more flexible semantic network participated in the current study. Given the theories relating processing of novel metaphors to flexible semantic memory structure (Kintsch, 2008; Faust, 2012), the present study supports the notion that creative ability and novel metaphor comprehension relate to semantic memory structure. However, future computational empirical studies that will directly link the relation between novel metaphor comprehension and a flexible semantic memory structure are needed.

The current study links individual differences in creative ability with metaphor comprehension via a semantic memory structure. Importantly, while higher creative individuals were significantly quicker in processing both conventional and novel metaphors, they exhibited only higher accuracy ratings for processing novel metaphors. Current neurocognitive theories on creativity focus on the role of executive functions in creativity (Benedek et al., 2014; Silvia, 2015), which can account for the shorter RTs exhibited by higher creative individuals. However, this cannot account for the accuracy effect found only for processing novel metaphors. This result, coupled with previous findings demonstrating how these individuals exhibit a more flexible semantic memory structure (Kenett et al., 2014), provides empirical support for the role of such a flexible semantic memory structure in novel metaphor processing. Furthermore, our findings support theories on metaphor comprehension that postulate semantic comparison and mapping in metaphor comprehension (Gentner, 1983; Bowdle and Gentner, 2005; Faust, 2012).

Our study has a few limitations. First, our results reveal that higher creative individuals were faster in processing all of the word-pair types compared to lower creative individuals. This may indicate that what differentiates the two groups are top-down, executive processes such as cognitive control mechanisms or fluid intelligence (Silvia, 2015). Nevertheless, our findings replicate previous findings (Gold et al., 2011). Furthermore, such an alternative interpretation cannot account for the differences in accuracy between the two groups, which were significant only for novel metaphors. Future studies are needed to further elucidate whether the relation between individual differences and creativity is due to differences in a semantic memory structure or executive processes. Furthermore, we examine how CoM differs at the aggregated, group level. Still, creativity is a true individual-based construct. Thus, future research is needed to examine the relation between creative ability and metaphor comprehension at the individual level.

## Conclusion

The current study examined the performance of lower and higher creative individuals on a metaphor comprehension task. Our findings provide further support for the role of a flexible semantic memory structure in contributing to semantic creativity, such as novel metaphors. Relating such high-level linguistic outputs to individuals that vary in creative ability sheds further light on these individuals and provides empirical support for linguistic theory on these semantic, creative, outputs.

## Author Contributions

Conception or design of the work: YK, RG, and MF. Data collection: YK.Data analysis and interpretation: YK, RG, and MF. Drafting the article: YK. Critical revision of the article: YK, RG, and MF. Final approval of the version to be published: YK, RG, and MF.

## Conflict of Interest Statement

The authors declare that the research was conducted in the absence of any commercial or financial relationships that could be construed as a potential conflict of interest. The handling Editor declared a past co-authorship, and shared affiliation, with two of the authors, YK and MF.

## Footnotes

1 www.jmp.com
